# Plasma Very‐Long‐Chain Fatty Acids in X‐Linked Adrenoleukodystrophy: Diagnostic Insights From a Clinical Laboratory Cohort

**DOI:** 10.1002/jcla.70269

**Published:** 2026-06-17

**Authors:** Sergio Molina Blas, Sergio Pérez Pujalte, Ana Moreno Álvarez, Ana Isabel Álvarez Ríos, Beatriz Muñoz Cabello, Constanza Navarro Moreno, Ana Lucia Gómez Gila, Elena Dios Fuentes, Eva Venegas Moreno, Carmen Delgado Pecellín

**Affiliations:** ^1^ Department of Clinical Biochemistry Hospital Universitario Príncipe de Asturias Madrid Spain; ^2^ Inherited Metabolic Disorders Unit, Department of Clinical Biochemistry Hospital Universitario Virgen del Rocío Seville Spain; ^3^ Department of Clinical Biochemistry Clínica Universidad de Navarra Pamplona Spain; ^4^ Department of Pediatrics Hospital Universitario Virgen del Rocío Seville Spain; ^5^ Department of Endocrinology Hospital Universitario Virgen del Rocío Seville Spain; ^6^ Asociación Española para el Estudio de los Errores Congénitos del Metabolismo (AECOM) Madrid Spain

**Keywords:** *ABCD1*, genotype–phenotype correlation, lipid metabolism, newborn screening, very long‐chain fatty acids (VLCFAs), X‐linked adrenoleukodystrophy (X‐ALD)

## Abstract

**Background:**

X‐linked adrenoleukodystrophy (X‐ALD) is a peroxisomal disorder caused by pathogenic variants in the *ABCD1* gene, leading to accumulation of very long‐chain fatty acids (VLCFAs). Plasma VLCFA measurement is central to diagnosis, but its value for prognostic stratification remains uncertain. This study explored the association between biochemical profiles, *ABCD1* variants, and clinical phenotypes, and assessed the clinical utility of plasma VLCFA levels.

**Methods:**

We performed a retrospective study of 31 genetically confirmed X‐ALD patients from Western Andalusia evaluated between 2005 and 2025. Clinical data, lipid profiles, and VLCFA parameters were analyzed. Patients were stratified by sex, clinical phenotype, genotype, and lipid status. Associations were assessed using Spearman correlation and non‐parametric tests, and ROC curves were used to evaluate discrimination for cerebral ALD (CALD).

**Results:**

Symptomatic patients showed higher C26:0 levels and C26:0/C22:0 ratios than asymptomatic individuals, with the highest values in patients with cerebral involvement. VLCFA levels differed across clinically defined groups, although substantial overlap was observed. Truncating ABCD1 variants were associated with higher C26:0 levels and C26:0/C22:0 ratios, but not with cerebral involvement. ROC analysis showed moderate discrimination for CALD (AUC = 0.84). No significant longitudinal changes in VLCFA levels were observed.

**Conclusions:**

Plasma VLCFA profiling remains essential for X‐ALD diagnosis and shows moderate group‐level associations with clinical phenotypes, particularly in cerebral involvement. However, its utility for individual prognostic stratification is limited, supporting the need for complementary biomarkers such as C26:0‐LPC.

## Graphical abstract

Plasma VLCFA levels are increased in symptomatic X‐linked adrenoleukodystrophy, particularly in patients with cerebral involvement. However, they do not reliably predict disease progression or longitudinal changes. These findings support their diagnostic value while highlighting the need for more robust prognostic biomarkers in clinical practice.

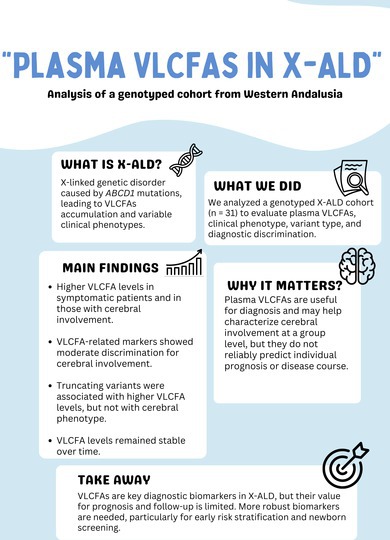

**Abbreviations**
*ABCD1*
: ATP binding cassette subfamily D member 1
ALD: Adrenoleukodystrophy
ALDP: Adrenoleukodystrophy protein
AMN: Adrenomyeloneuropathy
AUC: Area under the curve
BMI: Body mass index
C26:0‐LPC: C26:0‐lysophosphatidylcholine
CALD: Cerebral adrenoleukodystrophy
GC/EI‐SIM‐MS: gas chromatography coupled to mass spectrometry with electron impact ionization and detection in selected ion monitoring mode
HDL: High density lipoprotein
HSCT: Hematopoietic stem cell transplantation
IQR: Interquartile range
LDL: Low density lipoprotein
MRI: Magnetic resonance imaging
NBS: Newborn screening
PAI: Primary adrenal insufficiency
ROC: Receiver Operating Characteristic
RUSP: Recommended Uniform Screening Panel
VLCFAs: Very long chain fatty acids
X‐ALD: X‐linked adrenoleukodystrophy

## 1 Introduction

X‐linked adrenoleukodystrophy (X‐ALD) is the most common inherited peroxisomal disorder, with a global prevalence of 1 in 20,000–50,000 individuals [1]. It is characterized by progressive neurodegeneration and adrenal insufficiency caused by defective peroxisomal β‐oxidation of saturated very long‐chain fatty acids (VLCFAs), resulting from loss of function of the adrenoleukodystrophy protein (ALDP) [2].

X‐ALD is caused by pathogenic variants in the *ABCD1* gene (Xq28), which encodes ALDP, a peroxisomal transporter required for the degradation of VLCFAs with carbon chains longer than C22:0 [3]. Loss of ALDP function leads to systemic accumulation of VLCFAs, contributing to characteristic neuroendocrine manifestations such as primary adrenal insufficiency (PAI) and hypogonadism [4]. The disorder shows complete penetrance in males and clinical manifestations in more than 80% of affected females, who may develop neurological symptoms. In accordance with current international consensus guidelines, females with X‐ALD are classified as symptomatic or asymptomatic/presymptomatic based on their clinical presentation [5]. Importantly, longitudinal studies have shown no clear correlation between plasma VLCFA levels and clinical progression in females with X‐ALD, highlighting the limited prognostic value of these biomarkers in this subgroup [6].

To date, more than 3000 *ABCD1* variants have been identified, many of which are unique to individual families [7]. Although most variants result in complete loss of ALDP function, some preserve residual activity and are associated with milder clinical phenotypes [8]. Despite extensive genetic characterization, a consistent genotype–phenotype correlation remains elusive, even among members of the same family [9, 10]. Currently, 978 variants registered in the ALD Mutation Database are categorized as “pathogenic or likely pathogenic” [7].

Clinically, X‐ALD presents a broad phenotypic spectrum, including childhood cerebral ALD (CALD), adrenomyeloneuropathy (AMN), primary adrenal insufficiency (PAI), and asymptomatic cases, with overlapping phenotypes being common. CALD typically manifests between ages 3 and 10 with neurocognitive decline and inflammatory demyelination, whereas AMN usually appears in adulthood with spastic paraparesis and peripheral neuropathy [11].

X‐ALD is a well‐recognized cause of PAI in boys and should be systematically considered in cases of unexplained adrenal insufficiency [12], whereas cerebral involvement in females is considered rare. Typically, mineralocorticoid function is preserved due to the selective involvement of the zona fasciculata and reticularis [13].

The diagnosis of X‐ALD is often delayed due to its clinical heterogeneity. Biochemical testing remains the first‐line diagnostic approach and includes plasma quantification of VLCFAs, particularly docosanoic acid (C22:0), tetracosanoic acid (C24:0), hexacosanoic acid (C26:0), as well as their ratios (C24:0/C22:0 and C26:0/C22:0). Although VLCFA profiling has been historically regarded primarily as a diagnostic tool, several studies have reported associations between VLCFA‐related biomarkers, including C26:0 and C26:0‐lysophosphatidylcholine (C26:0‐LPC), and clinical severity or cerebral involvement. However, these associations have not been consistently observed across studies, and their prognostic value remains uncertain, likely reflecting the influence of additional modifiers [13, 14]. The definitive diagnosis is established by identifying pathogenic variants in the *ABCD1* gene through targeted sequencing, which confirms the molecular defect underlying the biochemical abnormalities [15].

In recent years, newborn screening (NBS) for X‐ALD has emerged as a valuable strategy for early detection and timely interventions such as hematopoietic stem cell transplantation (HSCT). X‐ALD was incorporated into the U.S. Recommended Uniform Screening Panel (RUSP) in 2016 [1]. In Europe, implementation remains heterogeneous, although some countries such as the Netherlands have already adopted NBS for X‐ALD [16]. In Spain, several regions have expanded their NBS programs to include X‐ALD, with Galicia among the first to incorporate it into extended metabolic panels [17]. In April 2026, X‐ALD was incorporated into the national NBS program as part of the common portfolio of services of the Spanish National Health System, with progressive implementation across regions aimed at achieving nationwide coverage.

Despite advances in molecular genetics and biochemical diagnostics, important gaps remain in understanding the relationship between VLCFA profiles, *ABCD1* variants, and clinical phenotypes. The present study was designed to explore the association between biochemical profiles, *ABCD1* variants, and clinical phenotypes in a cohort of patients with X‐ALD from Western Andalusia. In addition, we aimed to evaluate the diagnostic and clinical utility of plasma VLCFA levels and their relationship with specific clinical phenotypes, particularly cerebral involvement, in both male and female patients.

## 2 Methods

### 2.1 Study Population

This retrospective, observational study included 31 patients from Western Andalusia with X‐ALD evaluated at Virgen del Rocío University Hospital (Seville, Spain) between 2005 and 2025. Inclusion criteria were genetically confirmed X‐ALD and availability of clinical data. Patients without genetic confirmation were excluded.

Biochemical analyses were performed in the subgroup of patients with available plasma VLCFA measurements. Patients lacking biochemical data were retained for clinical and genetic characterization but were excluded from analyses requiring laboratory measurements.

Clinical and biochemical data were retrospectively reviewed to ensure temporal alignment between both datasets. Plasma VLCFA levels were analyzed in relation to the clinical phenotype documented at the time of sample collection.

Additional clinical variables, including age at diagnosis and symptom status at diagnosis, were collected when available to minimize misclassification over time.

### 2.2 Demographic and Clinical Characteristics

The cohort included 31 patients (17 males and 14 females) with genetically confirmed X‐ALD. Age at sampling ranged from 2 to 63 years (median 25 years). Of the 12 minors, 9 were boys and 3 were girls. Patients with CALD were younger (median 11 years), whereas AMN and PAI were observed in adults (median 38 and 31 years, respectively). The mean clinical follow‐up was 7.4 years (range 1–20 years).

### 2.3 Clinical Stratification

Patients were classified as symptomatic or asymptomatic based on the presence of clinical manifestations associated with X‐ALD. Symptomatic patients included those presenting with PAI, AMN, or CALD. Individuals with an *ABCD1* variant but without clinical or endocrine manifestations at the time of evaluation were classified as asymptomatic or presymptomatic, most of whom were identified through family screening.

Clinical phenotypes were defined based on widely accepted clinical features of X‐ALD. PAI was defined by low basal cortisol levels with elevated ACTH. AMN was defined as progressive spinal cord involvement characterized by spastic paraparesis in the absence of active cerebral inflammatory lesions. CALD was defined by neurological symptoms associated with inflammatory demyelinating lesions on brain MRI.

For analytical purposes, patients were grouped into asymptomatic/presymptomatic individuals, non‐cerebral symptomatic patients (PAI and/or AMN), and patients with cerebral involvement (CALD). This grouping was used to facilitate statistical comparisons in a relatively small cohort and does not represent a formal severity classification or imply a linear progression of the disease.

### 2.4 Lipid Profile and Hypercholesterolemia Definition

Hypercholesterolemia was defined as total serum cholesterol > 200 mg/dL (5.18 mmol/L) or the use of lipid‐lowering therapy in adults. In pediatric patients, age‐specific thresholds were applied according to current European guidelines.

Given that lipid‐lowering therapies may influence lipid metabolism and potentially affect VLCFA levels, their use was considered a potential confounding factor. However, patients receiving lipid‐lowering treatment (*n* = 2) were not excluded, as the study aimed to reflect real‐world clinical conditions. Both patients were on stable treatment for more than 6 months at the time of sampling.

Nine patients had received Lorenzo's oil during follow‐up, although its use was heterogeneous and not ongoing at the time of sampling. One pediatric patient with cerebral involvement had undergone gene therapy prior to referral, but detailed treatment data were unavailable. No other patients received disease‐modifying therapies such as HSCT.

### 2.5 Biochemical and Molecular Characterization

Plasma concentrations of VLCFAs, pristanic acid, phytanic acid, and standard lipid profiles were available for 29 of the 31 patients. The remaining two patients, recently diagnosed based on pathogenic ABCD1 variants, were included for clinical and genetic characterization but were excluded from analyses requiring biochemical measurements.

Blood samples were centrifuged at 3000 × g for 10 min, and plasma aliquots were stored and shipped on dry ice to the Center for the Diagnosis of Molecular Diseases (CEDEM, Madrid, Spain). VLCFAs, phytanic acid, and pristanic acid were quantified by gas chromatography–mass spectrometry using electron impact ionization and selected ion monitoring (GC/EI‐SIM‐MS; Agilent 5560/5977), following previously validated protocols for VLCFA quantification [3]. Samples were subjected to acid hydrolysis, followed by liquid–liquid extraction with hexane, and analyzed as fatty acid methyl esters. Reference ranges for VLCFA interpretation are detailed in Table 1.

**TABLE 1:** Demographic, clinical and biochemical characteristics of the study population.

Variable	Value, *n* (%) or median (IQR)
**Demographic characteristics**	
Age (years)	25 (16–48)
Age at diagnosis (years)	20 (6–42.5)
Sex	
Female	14 (45.16)
Male	17 (54.84)
Body mass index (kg/m^2^)	22.48 (17.88–25.77)
Clinical characteristics	
Asymptomatic	14 (45.16)
Symptomatic	17 (54.84)
Adrenal insufficiency (PAI)	10 (32.26)
Adrenomyeloneuropathy (AMN)	9 (29.03)
Cerebral ALD (CALD)	9 (29.03)
**Biochemical parameters**	
C22:0 (μmol/L)	45 (37.5–56.5) [*n* = 28]
C24:0 (μmol/L)	51.5 (40–61.84) [*n* = 28]
C26:0 (μmol/L)	1.12 (0.64–2.18) [*n* = 29]
C24:0/C22:0 ratio	1.13 (0.9–1.49) [*n* = 29]
C26:0/C22:0 ratio	0.029 (0.01–0.054) [*n* = 29]
Total cholesterol (mg/dL)	194 (160.5–236.5) [*n* = 27]
LDL cholesterol (mg/dL)	101 (70–152) [*n* = 25]
HDL cholesterol (mg/dL)	59 (52.5–71.5) [*n* = 27]
Triglycerides (mg/dL)	93 (67.5–132) [*n* = 27]

*Note:* Reference levels: C22:0 (50 ± 16 μmol/L); C24:0 (38 ± 14 μmol/L); C26:0 (0.55 ± 0.17 μmol/L); C24:0/C22:0 (0.77 ± 0.12); C26:0/C22:0 (0.012 ± 0.004). Data are presented as median (IQR) or *n* (%). Clinical phenotypes may overlap within individual patients. Due to the retrospective nature of the study, some variables were not available for all patients; therefore, the number of observations (*n*) is reported for each parameter.

Abbreviations: BMI, body mass index; C22:0, docosanoic acid; C24:0, tetracosanoic acid; C26:0, hexacosanoic acid; VLCFAs, very long‐chain fatty acids.

Lipid parameters—including total cholesterol, low‐density lipoprotein cholesterol (LDL‐C), high‐density lipoprotein cholesterol (HDL‐C), and triglycerides—were measured by spectrophotometry using a COBAS 8000 analyzer (Roche Diagnostics).

Diagnosis of X‐ALD was confirmed by targeted sequencing of the *ABCD1* gene, with variant classification based on ACMG/AMP guidelines.

### 2.6 Statistical Analysis

Statistical analysis was performed using GraphPad Prism (version 10.4.2). Data normality was assessed using the Shapiro–Wilk test. Continuous variables were compared between groups (sex, clinical status, and hypercholesterolemia) using the Mann–Whitney U test or Kruskal–Wallis test, as appropriate, followed by Dunn's multiple comparisons test for post hoc analysis. Associations between biochemical parameters, age, and lipid profile were evaluated using Spearman's correlation coefficient.

Receiver operating characteristic (ROC) curve analysis was performed to assess the ability of VLCFA‐related biomarkers (C26:0, C24:0, C26:0/C22:0, and C24:0/C22:0) to discriminate between patients with cerebral involvement (CALD) and those without cerebral involvement (including asymptomatic and PAI/AMN cases). The area under the curve (AUC) with 95% confidence intervals was calculated for each marker.

A two‐sided *p* < 0.05 was considered statistically significant.

The datasets used for statistical analyses and figure generation are provided in Table S3.

## 3 Results

### 3.1 Demographic and Clinical Characteristics

A total of 31 patients from Western Andalusia with genetically confirmed X‐ALD were included. Seventeen patients (54.8%) were male and 14 (45.2%) were female. Demographic and clinical characteristics are summarized in Table 1.

The median age was 25 years (IQR 16–48), and the median age at diagnosis was 20 years (IQR 6–42.5). Fourteen patients (45.2%) were asymptomatic at diagnosis, whereas 17 (54.8%) presented clinical manifestations. PAI was observed in 10 patients (32.3%), AMN in 9 (29.0%), and CALD in 9 (29.0%). Clinical phenotypes were not mutually exclusive and may overlap within individual patients. Detailed individual clinical, genetic, and biochemical data are provided in Table S1.

Among female patients, 5 of 14 (35.7%) were symptomatic, including 2 with CALD (one adult and one pediatric patient who died at 7 years of age; Patient 31). The remaining symptomatic females mainly exhibited AMN with spastic paraparesis, and one also showed PAI.

### 3.2 Biochemical Profile According to Sex and Clinical Presentation

When comparing biochemical parameters by sex, a statistically significant difference was observed in the C24:0/C22:0 ratio, which was higher in males (*p* = 0.0103). No significant differences were found in C26:0 levels (*p* = 0.618) or in the C26:0/C22:0 ratio (*p* = 0.136) between male and female patients.

Sex‐stratified analysis was performed in 17 males and 12 females, as two female patients lacked available VLCFA measurements. The distribution of VLCFA levels according to sex is illustrated in Figure S1.

When stratifying patients according to clinical status (symptomatic vs. asymptomatic), significant differences were observed in several VLCFA‐related markers. Symptomatic patients showed higher C26:0 levels (*p* = 0.007), C24:0/C22:0 ratio (*p* = 0.028), and C26:0/C22:0 ratio (*p* = 0.012). In contrast, no significant differences were found in pristanic acid, phytanic acid, C22:0, or C24:0 levels, nor in lipid profile parameters.

To further characterize these findings, VLCFA levels were compared across clinically defined patient groups, including asymptomatic individuals, non‐cerebral symptomatic patients (PAI and/or AMN), and patients with cerebral involvement (CALD).

### 3.3 Biochemical Profile Across Clinically Defined Patient Groups

**FIGURE 1:**
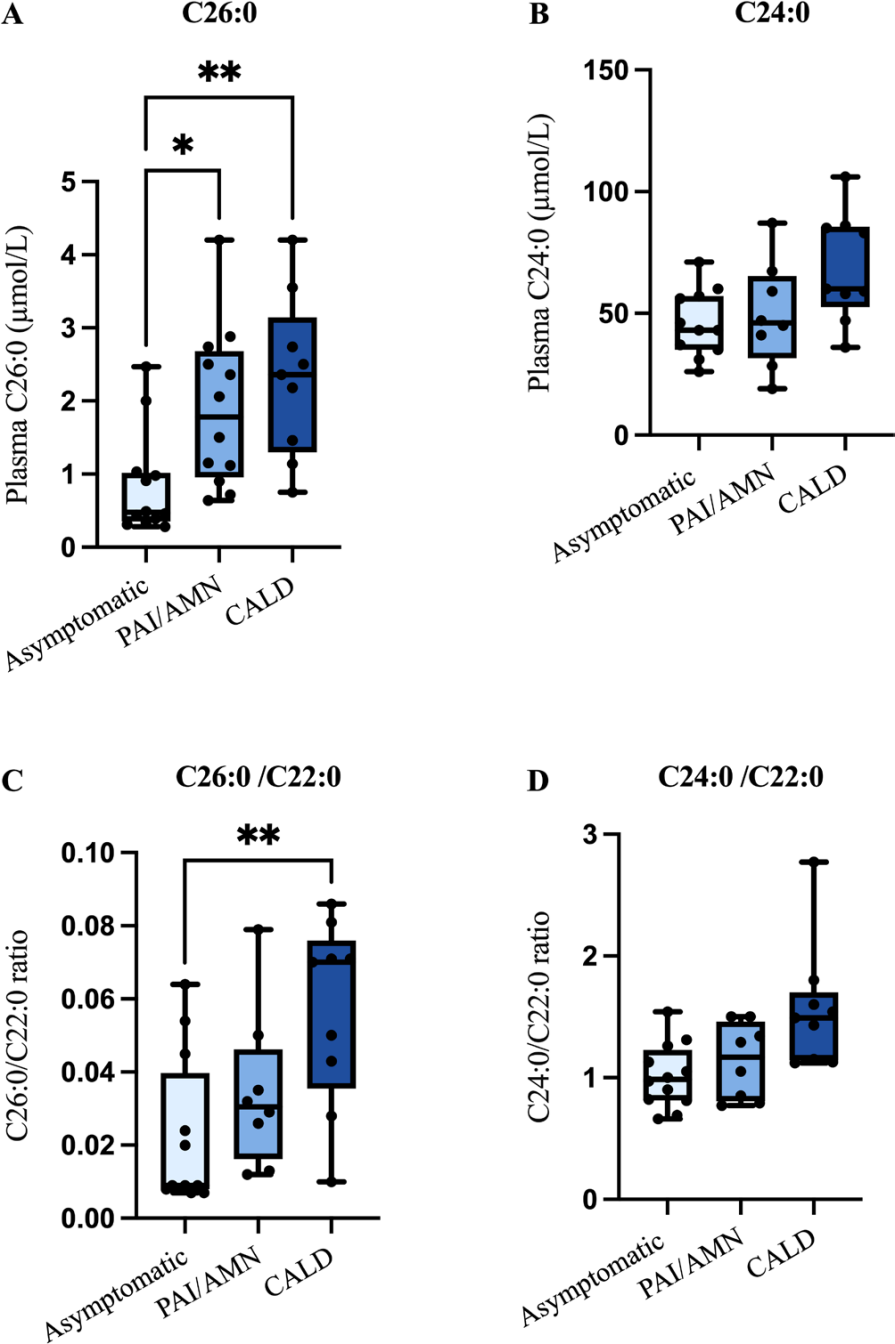
Plasma VLCFA levels across clinically defined patient groups. Box plots showing plasma C26:0, C24:0, and VLCFA ratios (C26:0/C22:0 and C24:0/C22:0) in asymptomatic patients, non‐cerebral symptomatic patients (PAI and/or AMN), and patients with cerebral involvement (CALD). Boxes represent the median and interquartile range (IQR); whiskers indicate minimum and maximum values. Group sizes were: Asymptomatic (*n* = 12), non‐cerebral symptomatic (*n* = 12), and CALD (*n* = 9). Statistical comparisons were performed using the Kruskal–Wallis test followed by Dunn's multiple comparisons test. Asterisks indicate statistically significant differences (*p < 0.05; **p < 0.01).

Plasma VLCFA levels were compared across clinically defined patient groups (Figure 1). C26:0 levels differed significantly between groups, with higher values observed in CALD compared to asymptomatic individuals (*p* < 0.01) and in non‐cerebral symptomatic patients compared to asymptomatic individuals (*p* < 0.05).

No significant differences were observed in plasma C24:0 levels between groups. Similarly, the C26:0/C22:0 ratio was significantly higher in CALD compared to asymptomatic individuals (*p* < 0.01), while no significant differences were detected between non‐cerebral symptomatic patients and the other groups. No significant differences were observed in the C24:0/C22:0 ratio across groups.

### 3.4 Association Between VLCFA Levels, Lipid Profile, and BMI

To further explore potential metabolic influences on VLCFA levels, we evaluated the relationship between VLCFA concentrations and parameters such as BMI and serum lipids.

The mean BMI in the cohort was 22.3 ± 5.47 kg/m^2^. No statistically significant correlations were found between BMI and VLCFA levels (*p >* 0.05).

**FIGURE 2:**
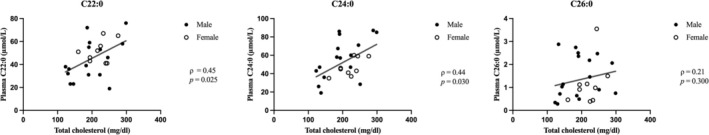
Association between total cholesterol and individual VLCFAs. Scatter plots showing the relationship between total serum cholesterol and plasma concentrations of C22:0, C24:0, and C26:0 in patients with X‐ALD. Male and female patients are represented by filled and open circles, respectively. Lines represent linear regression for visualization purposes. Spearman correlation coefficients (ρ) and *p* values are indicated in each panel. VLCFAs: Very long‐chain fatty acids; C22:0, docosanoic acid; C24:0, tetracosanoic acid; C26:0, hexacosanoic acid.

In contrast, hypercholesterolemia was observed in 51.6% of patients. Spearman correlation analysis revealed significant positive associations between total cholesterol and both C24:0 (ρ = 0.494, *p =* 0.012) and C22:0 (ρ = 0.518, *p =* 0.008), whereas no significant correlation was observed for C26:0 (ρ = 0.211, *p =* 0.302) (Figure 2). No correlations were found between VLCFA levels and HDL cholesterol or triglycerides (*p >* 0.05 for all).

When stratified by lipid status, hypercholesterolemic individuals had significantly higher C26:0 levels (*p =* 0.032), while no significant differences were observed for C24:0. These differences are illustrated in Figure S2.

### 3.5 Discriminative Potential of VLCFAs for Cerebral Involvement

A subanalysis was conducted in 9 patients with CALD (7 males, 2 females). Cerebral disease severity was evaluated using the Loes score, a semi‐quantitative MRI scale of demyelination. The mean Loes score in this subgroup was 4.25.

Hypercholesterolemia was present in 88.9% of CALD patients. However, no correlations were found between total cholesterol and Loes score (*p =* 0.201), nor between Loes score and VLCFA levels or lipid profile parameters.

ROC curve analysis was performed to assess the ability of VLCFA‐related biomarkers (C26:0, C24:0, C26:0/C22:0, and C24:0/C22:0) to discriminate between patients with CALD and those without cerebral involvement.

**FIGURE 3:**
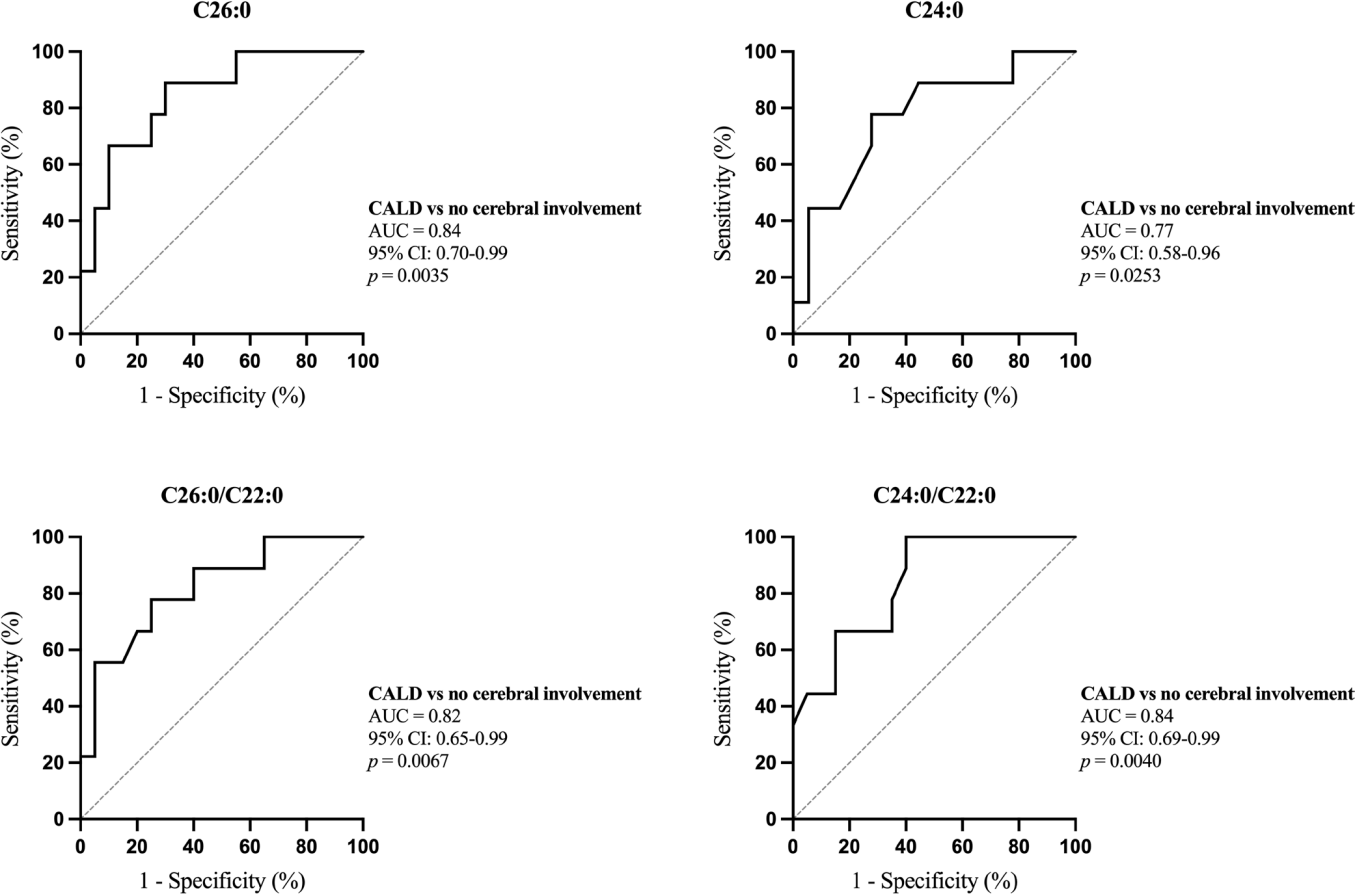
Diagnostic performance of plasma VLCFA‐related markers for cerebral involvement in X‐ALD. ROC curves showing the ability of C26:0, C24:0, C24:0/C22:0, and C26:0/C22:0 to discriminate between patients with CALD and those without cerebral involvement. The area under the curve (AUC), 95% confidence intervals, and *p* values are indicated in each panel. VLCFAs: Very long‐chain fatty acids; CALD: Cerebral adrenoleukodystrophy; ROC: Receiver operating characteristic.

C26:0 yielded an AUC of 0.84 (95% CI: 0.6959–0.9930; *p =* 0.0035), the C24:0/C22:0 ratio an AUC of 0.84 (95% CI: 0.6920–0.9858; *p =* 0.0040), and the C26:0/C22:0 ratio an AUC of 0.82 (95% CI: 0.6529–0.9860; *p =* 0.0067). C24:0 yielded an AUC of 0.77 (95% CI: 0.5763–0.9607; *p* = 0.0253) (Figure 3).

### 3.6 Genetic Analysis

Targeted sequencing identified 12 distinct *ABCD1* variants, which are provided in Table S2. Of these, 58.1% were missense, and 39.0% were truncating variants (frameshift or nonsense).

One case involved a 7‐year‐old female with the c.390dup (p.Gly131Trpfs*64) variant, previously reported in a newborn screening case without follow‐up [17]. This patient showed the highest VLCFA levels in the cohort (C26:0 = 4.2 μmol/L; C24:0 = 106 μmol/L; C26:0/C22:0 = 0.071; C24:0/C22:0 = 1.8) and developed a rapidly progressive neurological phenotype suggestive of childhood CALD. She was a female of Algerian origin with a confirmed 45X0 karyotype consistent with Turner syndrome and a pathogenic *ABCD1* variant. Early clinical features included dysmorphic characteristics and lymphedema from infancy. After normal early development, she developed progressive psychomotor regression from the age of four, characterized by gait impairment with progressive claudication, sialorrhea, rapid language deterioration leading to loss of speech, and adrenal insufficiency. Brain MRI revealed bilateral signal abnormalities involving deep structures and corticospinal tracts, suggestive of a metabolic or neurodegenerative process, and EEG showed disorganized activity with subclinical epileptiform features. She died within 1 year of symptom onset. Clinical data were limited, as the patient was referred from another country, and detailed longitudinal information was not available.

**FIGURE 4:**
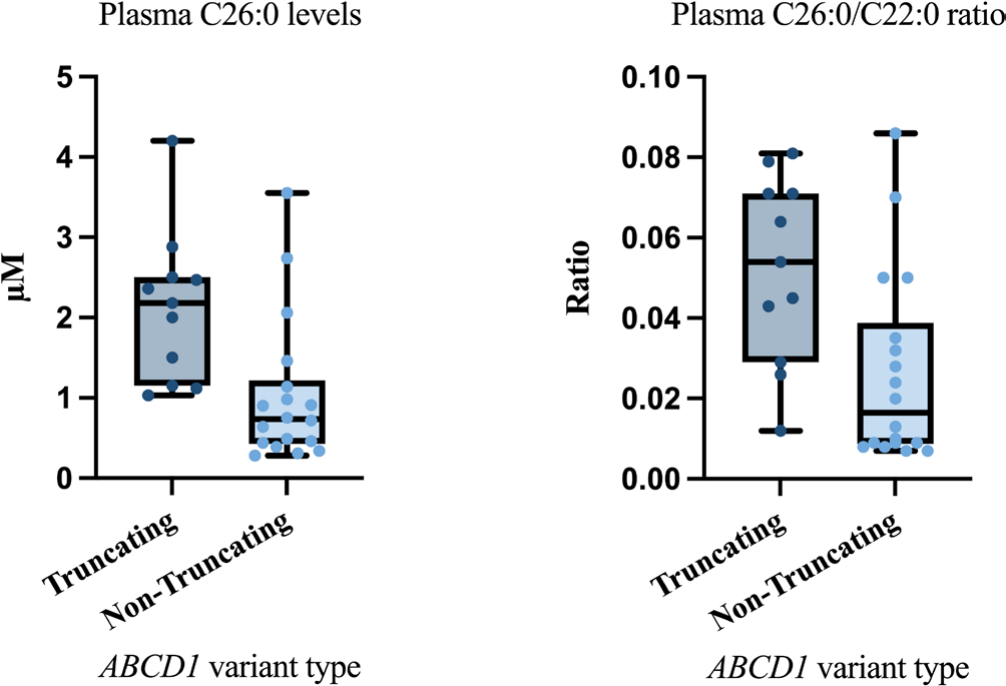
VLCFA levels according to *ABCD1* variant type. Box plots with individual data points showing plasma C26:0 concentrations and C26:0/C22:0 ratios in patients carrying truncating versus non‐truncating variants. Truncating variants were associated with higher VLCFA levels (*p* ≤ 0.0057; *n* = 29). VLCFAs: Very long‐chain fatty acids.

Patients with truncating variants had significantly higher C26:0 levels (median 2.18 μmol/L vs. 0.735 μmol/L; *p* = 0.0010) and higher C26:0/C22:0 ratios (median 0.0540 vs. 0.0165; *p* = 0.0057) than those with non‐truncating variants (Figure 4).

No association was found between variant type and cerebral involvement (Fisher's exact test, *p* = 1.000).

### 3.7 Longitudinal Stability of VLCFA Levels in X‐ALD Patients

Twelve patients had serial VLCFA measurements. Of these, 9 with measurements obtained at different time points were included in the longitudinal analysis (Figure S3). The average time between samples was 5.08 ± 6.13 years. Wilcoxon signed‐rank tests showed no significant differences between time points (*p* > 0.05).

When clinical phenotypes (PAI, AMN, and CALD) were analyzed separately, no significant longitudinal changes in plasma VLCFA levels were observed. Longitudinal evolution was assessed as the relative change (Δ%) between the first and last measurements. The mean variation (Δ%) was less than 10% for all markers, with no association with clinical status or disease duration (*p* > 0.05).

## 4 Discussion

In this study, we evaluated the diagnostic performance and clinical relevance of plasma VLCFA levels in a genotyped cohort of patients with X‐ALD. Historically, females were considered asymptomatic due to the X‐linked inheritance pattern [18]. However, Engelen et al. reported that more than 80% of females develop neurologic symptoms with increasing age [19]. Accordingly, current international guidelines recommend classifying female patients as symptomatic or asymptomatic/presymptomatic rather than using the terms “carrier” or “heterozygous” [5].

Although X‐ALD is a monogenic disorder, its clinical presentation is highly heterogeneous, even within the same family [20, 21]. In our cohort, intrafamilial phenotypic variability was observed despite shared genotypes, consistent with previous reports, including monozygotic twins [10]. This variability has been attributed to genetic, epigenetic, and environmental modifiers [22]. In our genotype–phenotype analysis, truncating variants were associated with higher C26:0 levels and C26:0/C22:0 ratios than non‐truncating variants, although no association with cerebral involvement was identified.

Although VLCFA accumulation is the hallmark biochemical defect in X‐ALD, its overall prognostic value remains limited. While some studies have reported associations between VLCFA‐derived biomarkers and clinical phenotypes [23, 24], more recent evidence has highlighted stronger correlations for alternative lipid biomarkers, such as C26:0‐LPC, particularly in relation to CALD [13, 14].

In our cohort, VLCFA levels differed across clinically defined patient groups, with the highest values observed in patients with cerebral involvement. During follow‐up, three patients with PAI developed neurological signs consistent with AMN, a phenomenon previously described in the literature. This finding highlights the need to interpret VLCFA levels within the clinical and temporal context, as noted by Jaspers et al. (2024) [25].

Group‐based comparisons across clinical phenotypes further supported these findings. In particular, C26:0 and the C26:0/C22:0 ratio were higher in patients with cerebral involvement, whereas no differences were observed for C24:0 or the C24:0/C22:0 ratio. Despite these group‐level differences, substantial overlap between phenotypes limited their discriminative utility. Accordingly, VLCFA levels should not be used for phenotypic stratification at the individual level. These findings support group‐level associations with specific clinical phenotypes, but not individual prognostic stratification, and reinforce the contribution of additional modifiers to disease expression.

An additional analysis exploring sex‐related differences in VLCFA levels revealed no significant differences in C26:0 concentrations or C26:0/C22:0 ratios between males and females. Although a statistically significant difference was observed in the C24:0/C22:0 ratio, this finding was not supported by consistent differences across other VLCFA‐related biomarkers and is unlikely to reflect a biologically meaningful sex‐specific alteration in VLCFA metabolism. These results should therefore be interpreted with caution and may be influenced by sample size and cohort‐specific variability.

While plasma VLCFA analysis, particularly C26:0, C24:0, and their ratios to C22:0, has a sensitivity above 99.9% in males, its diagnostic sensitivity in asymptomatic/presymptomatic females is notably lower (~85%) [26]. Recent studies have shown that measuring C26:0‐LPC in plasma or dried blood spots improves diagnostic accuracy, especially in females and in newborn screening settings [27, 28]. Accordingly, C26:0‐LPC should be considered the preferred biomarker for screening and diagnostic evaluation in females, as VLCFA analysis alone may be insufficient. In line with previous reports, females with pathogenic variants in the *ABCD1* gene typically show delayed and variable neurological involvement.

Another finding from our cohort is the association between plasma VLCFA levels and total cholesterol. Significant positive correlations were observed for C24:0 and C22:0, whereas C26:0 did not show a significant association, suggesting a differential relationship between individual VLCFAs and lipid metabolism. Although lipid‐lowering agents such as lovastatin have been shown to reduce VLCFA levels, they have not demonstrated clinical or radiological benefit [29, 30]. Exploratory analyses based on lipid status showed that only C26:0 remained significantly elevated in hypercholesterolemic individuals, likely reflecting the loss of information associated with dichotomizing continuous variables.

In the CALD subgroup, two female patients exhibited rapidly progressive cerebral involvement, supporting the need for clinical monitoring beyond genetic classification. Both showed the highest C26:0 levels in the cohort. One was a pediatric patient with a truncating *ABCD1* variant (c.390dup; p.Gly131Trpfs*64) who died within 1 year of symptom onset. Turner syndrome may have contributed to the severity of the clinical presentation by reducing the availability of a functional *ABCD1* allele. Although VLCFA levels were elevated in CALD, they are not reliable indicators of disease progression or central nervous system‐specific metabolic changes, and plasma concentrations may not fully reflect cerebral activity [31].

ROC analyses showed that C26:0 and related VLCFA ratios had moderate discriminative performance for identifying CALD at diagnosis, although substantial overlap between groups limits their utility at the individual level. These findings indicate that VLCFA‐related biomarkers may provide moderate discrimination for cerebral involvement at a group level, but they do not provide prognostic information regarding future neurological manifestations. As HSCT remains the only proven disease‐modifying therapy for CALD and is most effective when initiated early [5], these findings reinforce the importance of early identification through NBS. X‐ALD is already included in the U.S. RUSP, and newborn screening implementation is expanding internationally [32]. In Spain, X‐ALD was incorporated into the national NBS program in April 2026, with progressive implementation across regions expected to achieve nationwide coverage.

Although C26:0‐LPC improves sensitivity in females and newborns, it is not disease‐specific and may also be elevated in other peroxisomal disorders such as Zellweger spectrum disorders, D‐bifunctional protein deficiency, or acyl‐CoA oxidase deficiency [33, 34]. Therefore, *ABCD1* sequencing remains the gold standard for definitive diagnosis.

This study has several limitations. First, the small sample size restricts statistical power, particularly within specific clinical subgroups, and the retrospective design introduces potential biases. Additionally, the absence of newer biomarkers, such as C26:0‐LPC or inflammatory markers, limits the extrapolation of our findings to central nervous system pathology. Plasma VLCFA levels may not fully reflect cerebral disease activity due to the compartmentalized nature of inflammatory demyelination in X‐ALD.

The retrospective design may also limit the precise temporal alignment between clinical status and biochemical measurements; although efforts were made to interpret VLCFA levels according to the phenotype at the time of sampling, residual discrepancies cannot be fully excluded. Furthermore, most analyses were based on cross‐sectional comparisons, limiting conclusions regarding temporal clinical evolution.

Neurological involvement was not assessed using standardized clinical scales (e.g., AACS or EDSS), which may reduce the precision of phenotypic classification. In addition, X‐chromosome inactivation patterns were not evaluated, limiting the interpretation of phenotypic variability in female patients.

The potential influence of lipid‐lowering therapies on VLCFA levels cannot be completely excluded. Although only two patients were receiving treatment at the time of sampling, this may have introduced residual confounding. Similarly, the heterogeneous use of Lorenzo's oil and the presence of a patient previously treated with gene therapy represent additional sources of variability.

Future prospective studies with larger cohorts, standardized clinical assessments, and integration of advanced lipidomic biomarkers are needed to validate and expand these findings.

## 5 Conclusion

In conclusion, our findings confirm the diagnostic value of plasma VLCFA profiling in X‐ALD and demonstrate group‐level associations with clinical phenotypes, particularly in patients with cerebral involvement. However, due to the substantial overlap between clinical phenotypes and the stability of VLCFA levels over time, their utility for individual prognostic stratification is limited. More sensitive biomarkers, such as C26:0‐LPC, together with early identification through NBS, may improve risk assessment and clinical management.

## Funding

The publication of this article was supported by the Asociación Española para el Estudio de los Errores Congénitos del Metabolismo (AECOM).

## Ethics Statement

The study was approved by the Research Ethics Committee of Hospitales Universitarios Virgen Macarena–Virgen del Rocío, Seville, Spain (session 12/2025, approved on December 16, 2025; study code 2025/2; SICEIA‐2025‐001752). Given the retrospective design and the use of anonymized clinical and laboratory data, informed consent was waived by the ethics committee. All procedures were conducted in accordance with the ethical standards of the institutional and national research committee and with the 1964 Helsinki Declaration and its later amendments.

## Consent

Waived by the ethics committee due to the retrospective design and use of anonymized data.

## Conflicts of Interest

The authors declare no conflicts of interest.

## Supporting information


**Figure S1:** Sex‐stratified distribution of VLCFA levels in X‐ALD patients. Box plots showing C26:0 levels (μmol/L), C24:0/C22:0 ratio, and C26:0/C22:0 ratio in male (*n* = 17) and female (*n* = 12) patients, with individual data points overlaid. The central line represents the median and boxes indicate the interquartile range (IQR); whiskers represent the full data range. *p* values were calculated using the Mann–Whitney U test. Two female patients were excluded due to missing VLCFA data.

**Figure S2:** Plasma VLCFA levels according to lipid status. (A) Plasma C26:0 levels and (B) plasma C24:0 levels in normocholesterolemic and hypercholesterolemic individuals. Data are presented as box plots with individual data points. Boxes represent the median and interquartile range (IQR); whiskers indicate minimum and maximum values. Statistical comparisons were performed using the Mann–Whitney U test. *p < 0.05. Total sample size: *n* = 27.

**Figure S3:** Longitudinal trajectories of plasma VLCFA levels. (A) Plasma C26:0 levels and (B) C26:0/C22:0 ratio over time in patients with serial measurements. Each line represents an individual patient. Time is expressed as years from the first available measurement. Twelve patients had serial measurements; nine with data from different time points were included in the analysis.

**Table S1:** Individual demographic, clinical, biochemical, and genetic characteristics of patients with X‐ALD included in the study cohort. Variables include sex, age at diagnosis, clinical phenotype, *ABCD1* variant, VLCFA parameters, and age at last follow‐up.

**Table S2:** jcla70269‐sup‐0005‐TableS2.csv. *ABCD1* variants identified in the study cohort, including genomic nomenclature, variant type, predicted molecular consequence, and associated clinical phenotypes.

**Table S3:** Datasets used for statistical analyses and figure generation, including raw VLCFA measurements, lipid parameters, and group stratifications used for comparative and correlation analyses.

## Data Availability

The datasets supporting the findings of this study are partially available within the article and its Supporting Information. Additional data are not publicly available due to privacy restrictions but are available from the corresponding author on reasonable request.
