# Supplementary figures and images for: Plasma Very‐Long‐Chain Fatty Acids in X‐Linked Adrenoleukodystrophy: Diagnostic Insights From a Clinical Laboratory Cohort
Source: J Clin Lab Anal. 2026 Jun 17;40(14):e70269. doi: 10.1002/jcla.70269 (PMC13399760; doi:10.1002/jcla.70269)

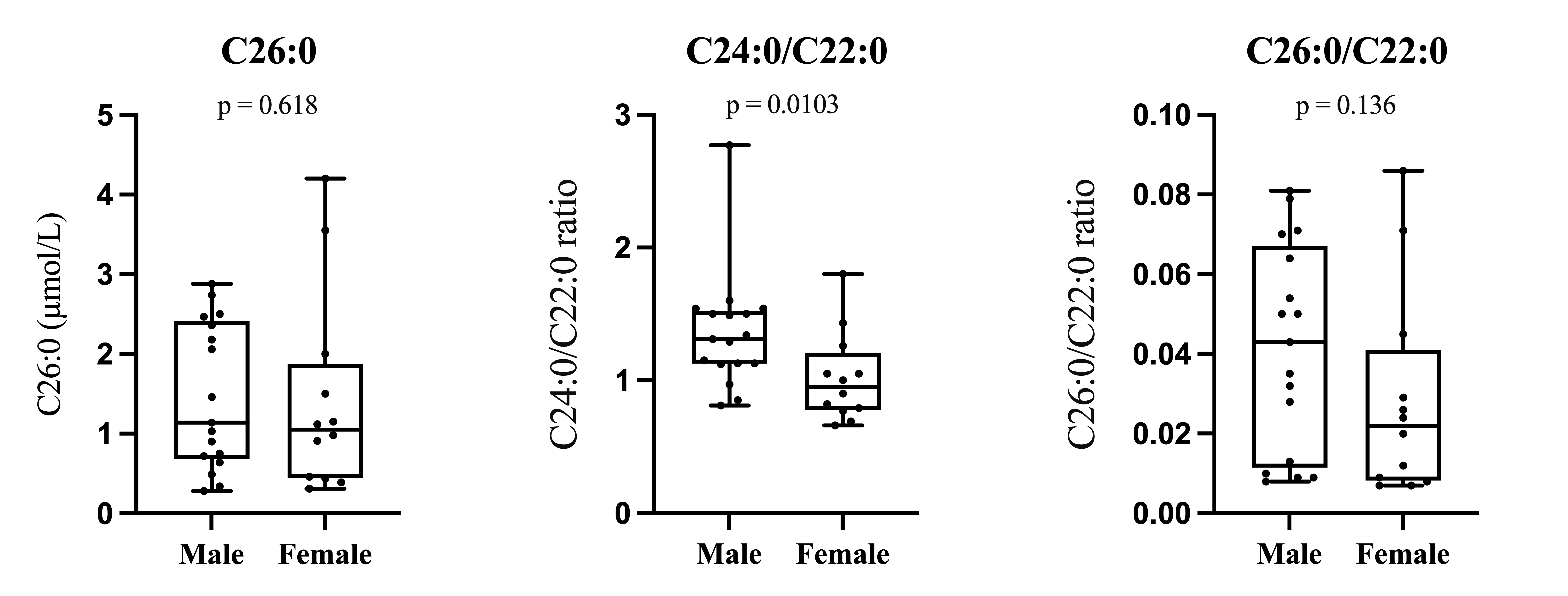

Supplement: Supplementary file 1 — Figure S1: Sex‐stratified distribution of VLCFA levels in X‐ALD patients. Box plots showing C26:0 levels (μmol/L), C24:0/C22:0 ratio, and C26:0/C22:0 ratio in male (n = 17) and female (n = 12) patients, with individual data points overlaid. The central line represents the median and boxes indicate the interquartile range (IQR); whiskers represent the full data range. p values were calculated using the Mann–Whitney U test. Two female patients were excluded due to missing VLCFA data. [file JCLA-40-e70269-s004.tiff]

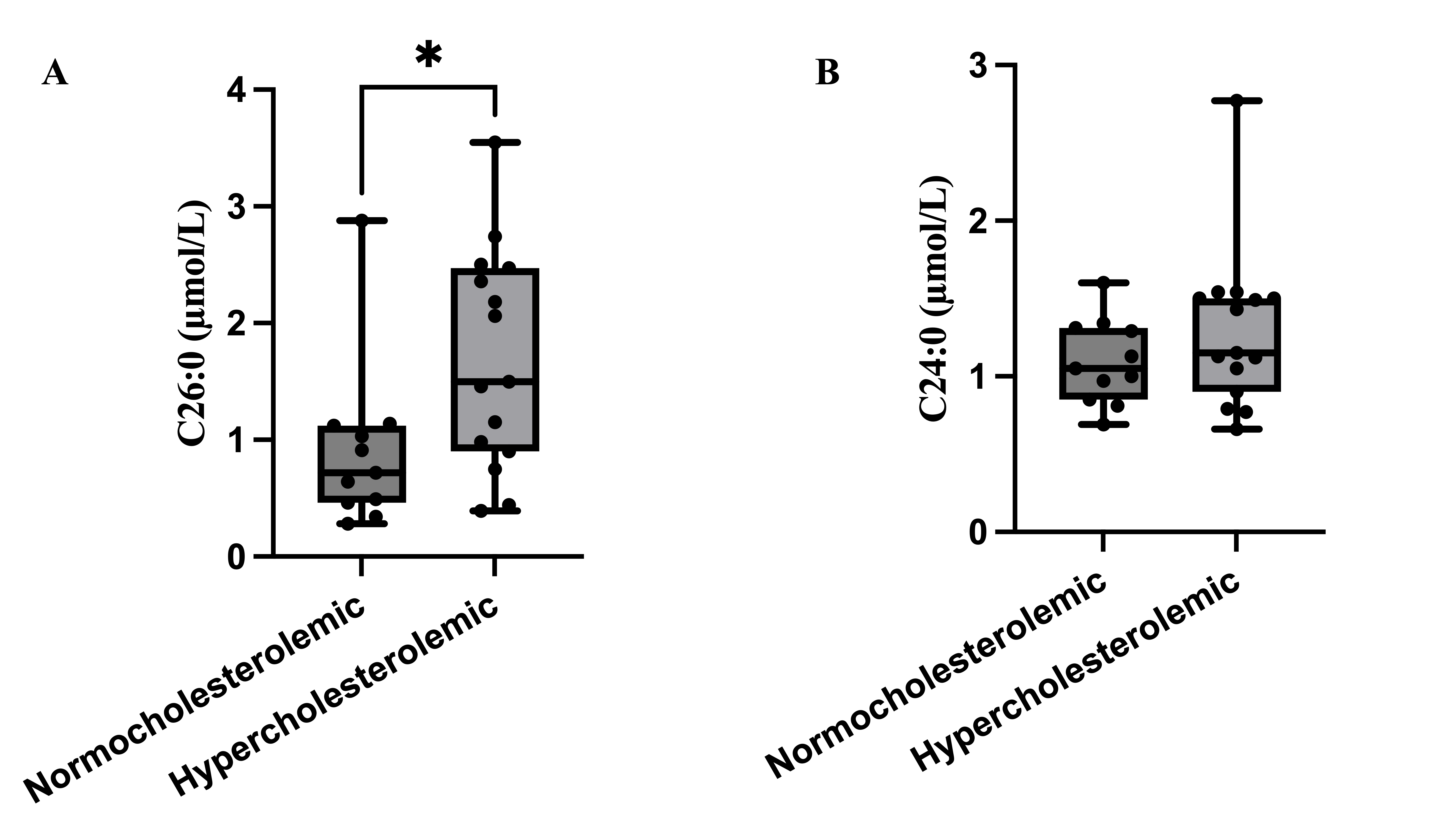

Supplement: Supplementary file 2 — Figure S2: Plasma VLCFA levels according to lipid status. (A) Plasma C26:0 levels and (B) plasma C24:0 levels in normocholesterolemic and hypercholesterolemic individuals. Data are presented as box plots with individual data points. Boxes represent the median and interquartile range (IQR); whiskers indicate minimum and maximum values. Statistical comparisons were performed using the Mann–Whitney U test. *p < 0.05. Total sample size: n = 27. [file JCLA-40-e70269-s002.tiff]

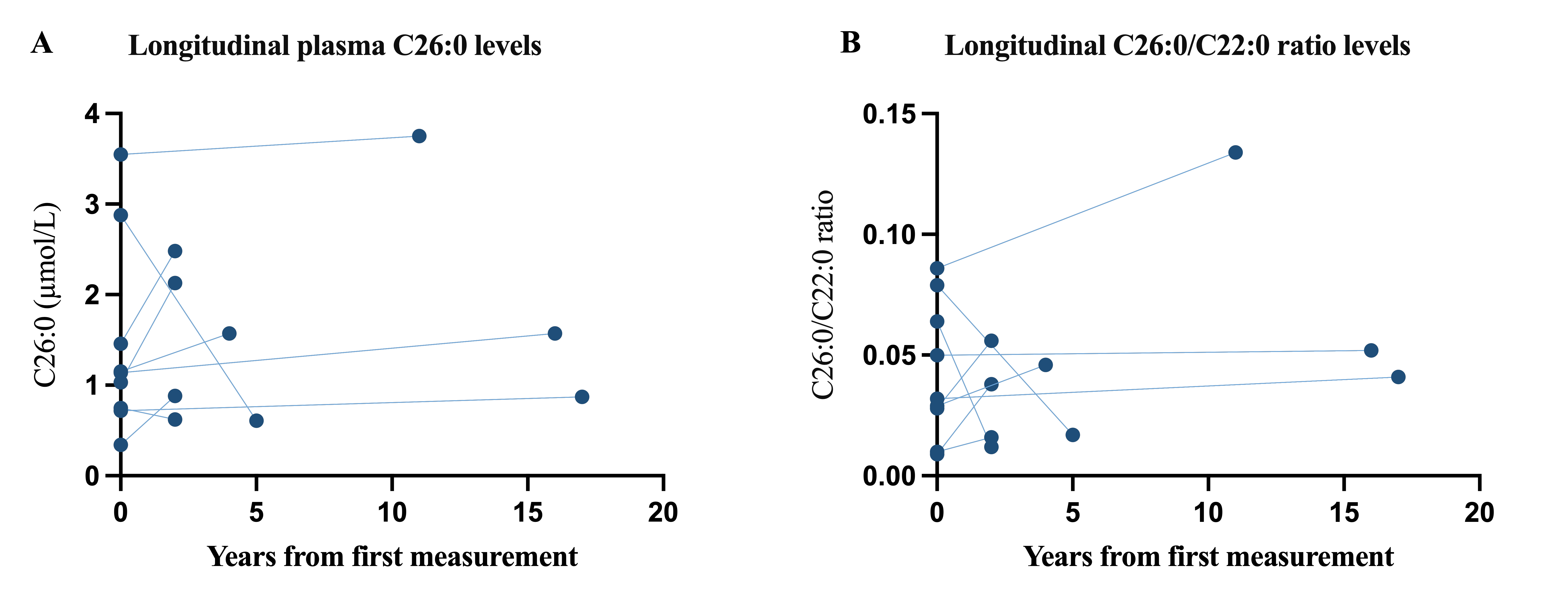

Supplement: Supplementary file 3 — Figure S3: Longitudinal trajectories of plasma VLCFA levels. (A) Plasma C26:0 levels and (B) C26:0/C22:0 ratio over time in patients with serial measurements. Each line represents an individual patient. Time is expressed as years from the first available measurement. Twelve patients had serial measurements; nine with data from different time points were included in the analysis. [file JCLA-40-e70269-s006.tiff]
